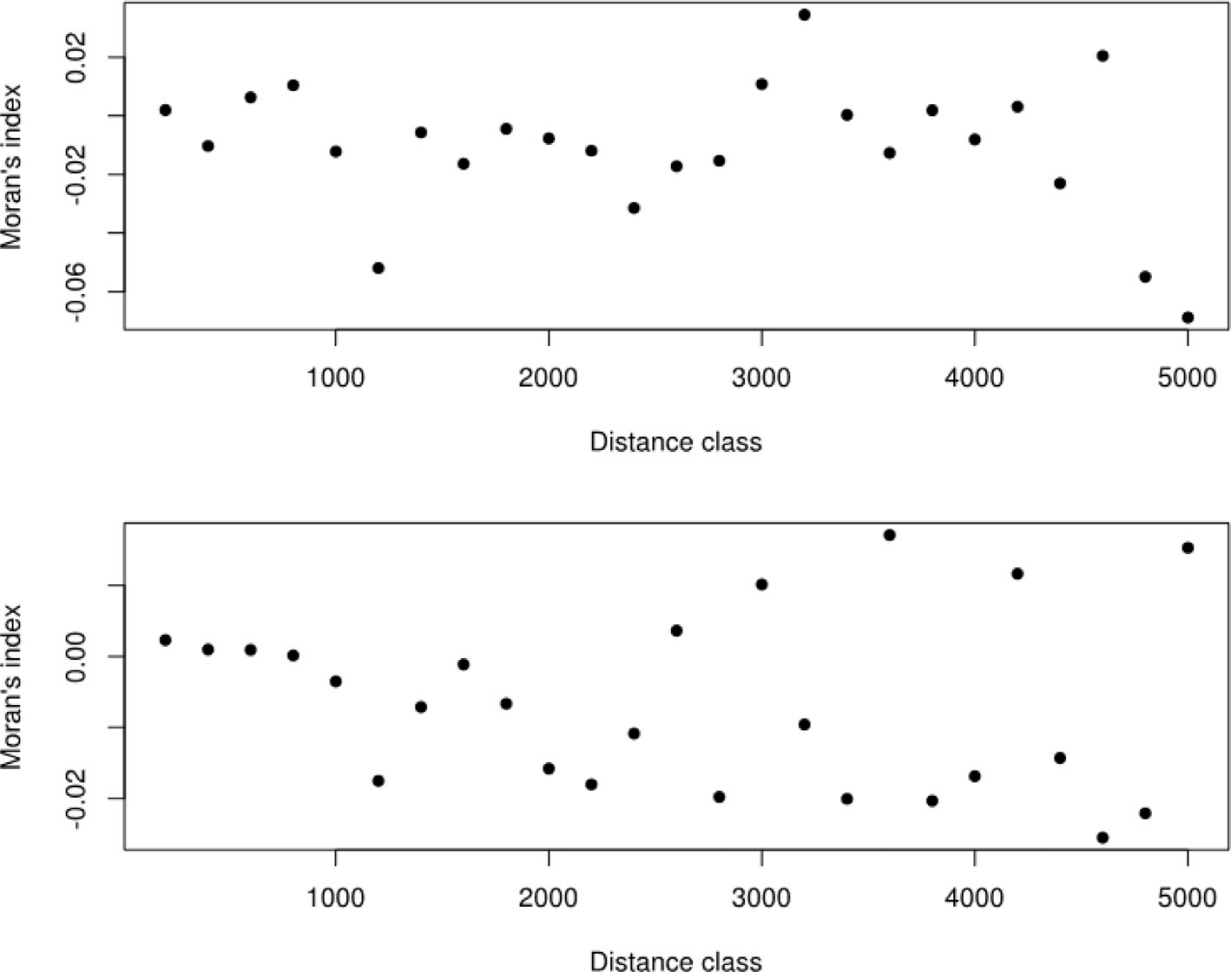# Identifying drivers of fox and cat faecal deposits in kitchen gardens in order to evaluate measures for reducing contamination of fresh fruit and vegetables

**DOI:** 10.1016/j.fawpar.2018.e00034

**Published:** 2018-12-29

**Authors:** M. Bastien, A. Vaniscotte, B. Combes, G. Umhang, V. Raton, E. Germain, I. Villena, D. Aubert, F. Boué, M.-L. Poulle

**Affiliations:** a University of Reims Champagne-Ardenne, SFR Cap Santé, EA 7510 ESCAPE, 51092 Reims cedex, France; b University of Reims Champagne-Ardenne, CERFE, 08240 Boult-aux-Bois, France; c French Establishment for Fighting Zoonoses (ELIZ), Domaine de Pixérécourt, 54220 Malzéville, France; d EcoDataDesign, 25000 Besançon, France; e ANSES, Nancy Laboratory for Rabies and Wildlife, National Reference Laboratory for *Echinococcus* spp., Wildlife Eco-epidemiology and Surveillance Unit, 54220 Malzéville, France; f CROC, Carnivore Research and Observation Center, 57590 Lucy, France; g University Hospital of Reims, Department of Parasitology-Mycology, National Reference Center for Toxoplasma, 51092 Reims cedex, France

**Keywords:** Environmental contamination, Foodborne parasites, *Echinococcus multilocularis*, *Toxoplasma gondii*, *Toxocara* sp.

## Abstract

Preventing foodborne pathogen contamination of raw fruit and vegetables in the field is critically important for public health. Specifically, it involves preventing faecal deposit by wildlife or domestic animals in fields of crops and kitchen gardens. The present study aims to identify the drivers of fox, dog and cat faecal deposits in kitchen gardens in order to mitigate the risk of contamination of raw produce with parasites shed in carnivore faeces. The focus was on *Echinococcus multilocularis*, ranked highest in the importance of foodborne parasites in Europe, but attention was also paid to other parasites of major concern - *Toxoplasma gondii* and *Toxocara* spp. During the winters of 2014 to 2016, faecal samples were collected from 192 kitchen gardens located in north-eastern France. From these samples, 77% contained scat of carnivores. Molecular analyses revealed that 59% of the 1016 faeces collected were from cats, 31% from foxes, and 10% from dogs. The ease of accessibility to kitchen gardens, the presence of food in the vicinity, and the composition of the surrounding vegetation were used to explain the distribution of fox and cat faeces. Generalized Linear Mixed Effects modelling showed that: i) fencing was not efficient in reducing cat faecal deposits, but drastically decreases those of foxes; ii) the abundance of *Microtus* sp. indicates a reason for the presence of both fox and cat faecal deposits, iii) the abundance of *Arvicola terrestris*, the proximity of fruit trees or farms and the predominance of forest and grassland around the village are all drivers of fox faecal deposits. These results point to the importance of fencing around kitchen gardens located in *E. multilocularis* endemic areas, particularly those surrounded by forest and grassland or close to fruit trees or farms.

## Highlights

• 192 kitchen gardens sampled 6 times over 2 winters, 1016 carnivore faeces collected.
• Fencing, food and vegetation cover tested as explanatory factors for faeces location.
• Numerous cat faeces, even in enclosed gardens. *Microtus* sp. drives their deposit.
• Garden accessibility, food and vegetation cover drive the faecal deposits of foxes.
• Fencing off kitchen gardens should be encouraged in *E. multilocularis* endemic areas.

## 1 Introduction

Fresh fruit and vegetables that are consumed either raw or have been minimally processed are increasingly recognized as transmission pathways for zoonotic foodborne pathogens (Robertson and Gjerde, 2001; Berger et al., 2010). Preventing field-contamination of these products is crucial in protecting public health. One challenging issue is the lack of a decontamination process for fresh fruits and vegetables eaten raw, where most of them undergo no “kill-step” from harvest to consumer (Alegbeleye et al., 2018; Jay-Russell, 2013). In the USA, traceability investigations conducted following *Escherichia coli* outbreaks in humans concluded that faecal deposits from feral swine and deer provided transmission pathways in locations where ready-to-eat baby spinach, strawberries and apple devoted to unpasteurised juice were grown (Cody et al., 1999; Jay et al., 2007; Laidler et al., 2013). These investigations highlight the fact that faecal deposits on plants from wild or feral animals carry a significant contamination risk of *E. coli* in pre-harvest produce (Cooley et al., 2007). As a consequence, the American produce industry published an agricultural ‘best practice’ document, specifically addressing bacterial contamination from ruminant and porcine species' faecal material deposited in or around crop fields and orchards (Jay-Russell, 2013). From this industrial context, very little attention has been paid to the risk of foodborne pathogen contamination from wild and feral animal faeces in domestic kitchen gardens. Given the current, indeed global emergence of *Echinococcus multilocularis* as a foodborne parasite (Davidson et al., 2012) consideration should be given to the risks associated with such deposits in locations where fruit and vegetables are grown.

*Echinococcus multilocularis* is responsible for human alveolar echinococcosis (AE), a rare but life-threatening disease, considered as one of the most dangerous helminthic zoonosis in the northern hemisphere (Conraths and Deplazes, 2015). Unless treated, this infection in humans is fatal. It is caused by ingesting infective eggs. The main challenge in preventing the spread of AE is educating the public and public health policy makers in the need for simple protective actions via a consistent message about contamination risk factors (Davidson et al., 2012). The good news is that preventive strategies against AE may also be effective against other foodborne zoonoses with similar transmission route, like toxocarosis and oocyst-induced toxoplasmosis. These zoonoses, respectively caused by *Toxocara* spp. and *Toxoplasma gondii*, are generally asymptomatic. However, in humans toxoplasmosis can cause miscarriage and disabling disease in fetuses, neonates, and immuno-compromised individuals (Robert-Gangneux et al., 2015), and toxocarosis can cause visceral *larva migrans* syndrome with potential ocular lesions (Overgaauw and van Knapen, 2013). As for *E. multilocularis*, the free-living infectious stages of these parasites are spread into the environment with faeces from the definitive host species. The red fox (*Vulpes vulpes*) is the main definitive host for *E. multilocularis* in Europe (Eckert and Deplazes, 2004), but the domestic dog (*Canis familiaris*) can also act as a definitive host (Deplazes et al., 2004; Umhang et al., 2012, Umhang et al., 2014). The domestic cat (*Felis s. catus*) can harbour *E. multilocularis* adults in its intestines leading to production of eggs (Knapp et al., 2016a; Umhang et al., 2015) but its contribution to environmental contamination seems to be negligible (Kapel et al., 2006; Hegglin and Deplazes, 2013). However, the domestic cat is the main *T. gondii* definitive host, with a single cat in its first infection being able to excrete millions of *T. gondii* oocysts in their faeces (Dubey, 2004). *Toxoplasma gondii* oocysts are most often excreted by kittens (Simon et al., 2018). Lastly, foxes, dogs and cats all serve as definitive hosts for *Toxocara* spp. (Baneth et al., 2016).

Several epidemiological studies identified the consumption of unwashed raw fruit and vegetables as a risk factor for AE (Kern et al., 2004; Piarroux et al., 2013), toxoplasmosis (Alvarado-Esquivel et al., 2011; Liu et al., 2009) and toxocarosis (Gyang et al., 2015; Fallah et al., 2016). The risk of human exposure to foodborne parasites appeared notable in privately owned kitchen gardens in north-eastern France where the density of fox and cat faeces is high (Bastien et al., 2018). In this same region, another study had emphasized that some kitchen gardens are at a high risk of contamination because, of the faecal deposits tested, most of them yielded positive qPCR results for *E. multilocularis* and *Toxocara* spp. DNA (Poulle et al., 2017).

Regarding these findings, the present study aims to identify the drivers of fox, dog and cat faecal deposit in kitchen gardens in north-eastern France to help draw species-specific measures which prevent foodborne parasite contamination in locations where fresh produce is grown. The focus was on fox faeces as main responsible of *E. multilocularis* environmental contamination, but attention was also paid to cat and dog faeces as potential carriers of foodborne parasites. The kitchen garden accessibility, the availability of food resources in the immediate vicinity, as well as the composition of the surrounding landscape, were tested as explanatory variables of carnivore faeces distribution in kitchen gardens.

## 2 Material and methods

### 2.1 Study area

**Fig. 1:**
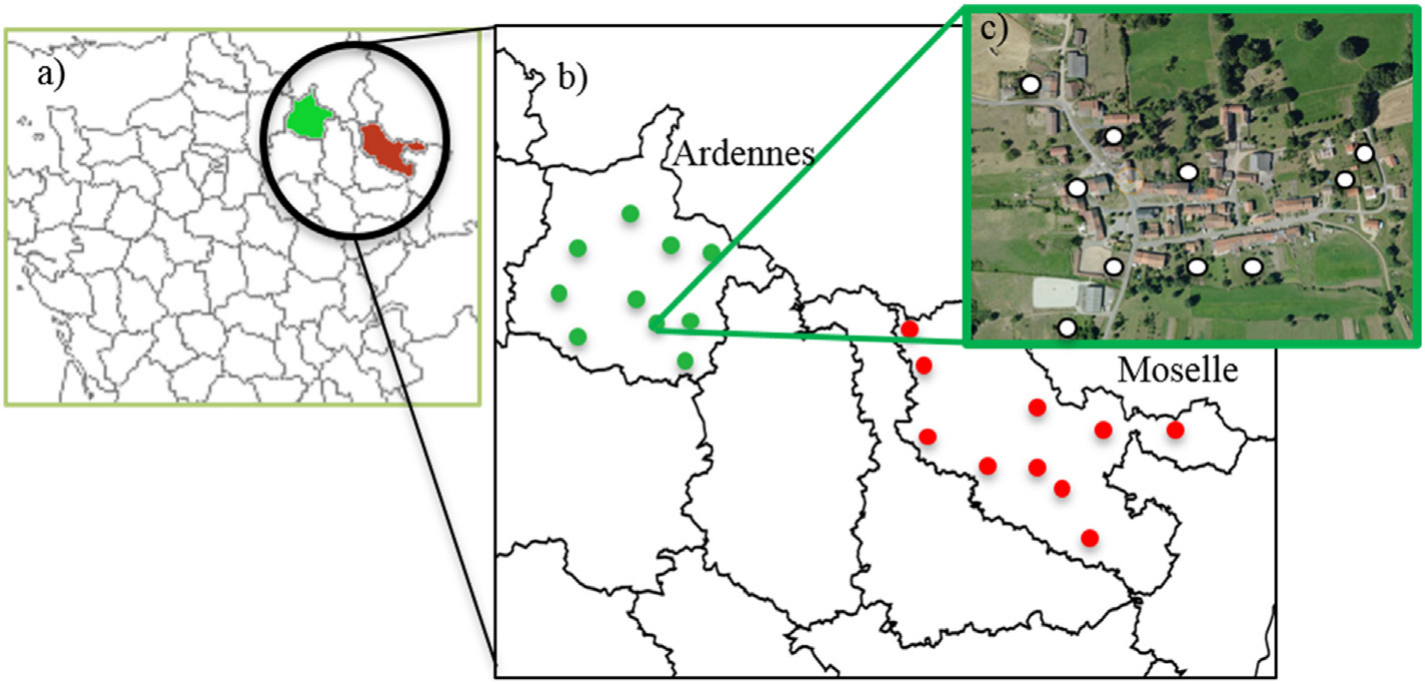
Localization of the two study areas (Ardennes and Moselle) in France (a), the prospected villages in Ardennes and Moselle regions (b), and the kitchen gardens sampled in one village (c).

The study took place in the French Ardennes (49° 25′ N, 4° 50′ E) and the Moselle (48° 49′ N, 6° 30′ E) regions, both located in north-eastern France (Fig. 1a) and among the French regions with the highest AE incidence (Piarroux et al., 2015). The Ardennes area is wooded (oak *Quercus* spp., beech *Fagus sylvatica*, hornbeam *Carpinus betulus* and spruce *Picea abies*), with cultivated fields and pastures and a low human population density (around 16 inhabitants/km^2^) with most of the villages having fewer than two hundred inhabitants. The fox density was estimated at about 3–4 foxes/km^2^ during the 2003–2006 period (Guislain et al., 2007) and did not significantly vary from 2004 to 2015 (Bastien et al., 2018). The cat population reached 142 individuals in a 460-ha area (~30 cats/km^2^) that encompassed two villages of the study area during the 2008–2010 period (Forin-Wiart et al., 2014), in other words approximately ten times that of the red fox population. The dog population did not exceed 30 individuals in these two villages (Poulle, pers. obs), so was at least four times smaller than the cat population. Meadow voles (*Microtus* sp.) and fossorial water voles (*Arvicola terrestris shermann*) are the main prey of both red foxes (Guislain et al., 2008) and domestic cats (Forin-Wiart, 2014) in this area. The Moselle region interchanges between wooded and industrialized areas with higher human density (around 170 inhabitants/km^2^) where villages comprise of approximately 1000 inhabitants. There were no stray dogs in the study area, while most of the domestic cats were free-ranging. *Echinococcus multilocularis* prevalence in the vulpine population is about 35% in Ardennes and Moselle regions (Combes et al., 2012).

### 2.2 Sampling design and molecular identification of the faeces shedder

Ten villages were selected in each region to ensure: i) the entire study area was covered (Fig. 1b); ii) A colleague served as an intermediary in the village to introduce us to local residents for confidence, authorization and support in obtaining samples. In each of the 20 villages selected (Fig. 1b), a 5-km buffer zone was defined from the village centre, that may include other villages. Four to thirteen gardens where fresh fruit and vegetables are cultivated were then chosen per buffer (Fig. 1c), depending on the size of the ‘social network’ of our local intermediary. As a result, a total of 192 of such locations were sampled in 38 villages, and the dimension of each was recorded. Of the 192 sampled sites, 185 were kitchen gardens devoted to household consumption; they were 207.3 ± 14.0 m^2^ in average size (min = 4 m^2^, max = 1276 m^2^). The other seven locations were larger cultivated areas devoted to market gardening, and spanning 7550.2 ± 2275.2 m^2^ on average (min = 512.5 m^2^, max = 20,553 m^2^). Both are later called ‘kitchen gardens’.

Sampling consisted of visual scans performed by walking the whole surface of the 192 kitchen gardens to detect and collect carnivore faeces. From January 2014 to December 2015, kitchen gardens were sampled every six weeks during October to March (i.e. out of the gardening period to avoid damaging the seedlings), totaling eight scans per kitchen garden. In Moselle, the snow cover prevented finding any scat during January 2015 but a further sample was conducted in January 2016.

All collected faeces were decontaminated over seven days at −80 °C and stored at −20 °C before being tested to determine the source animal. A 0.5 g of each copro-sample was treated for DNA extraction using the QIAamp Fast DNA Stool Mini kit (Qiagen, Hilden, Germany) following manufacturer recommendations. The carnivorous species (fox, cat or dog) was then identified using a multiplex real-time PCR assay following Knapp et al. (2016b).

### 2.3 Kitchen garden attributes assumed to explain their use by foxes

#### 2.3.1 Kitchen garden accessibility

The accessibility of kitchen gardens was categorized as ‘open’ (no fencing or walls around them), ‘enclosed’ (with a continuous fence or wall at least 1.2 m high), or ‘partially open’ (with a non-continuous fence or wall). Of the 192 kitchen gardens, 60 were open, 40 were enclosed and 92 were partially open. In total, 45.7 ha were sampled 8 times and 60% of this surface area made up open kitchen gardens, whereas 13% were enclosed and 27% partially open.

#### 2.3.2 Presence/absence of food resources in the immediate vicinity

Food resources for fox and habitats favorable to fox prey were considered as a potential lure for this canid, and therefore potential drivers for their faecal deposits. Thus, the presence/absence of fruit trees (indicator of the presence of fruits in the ground), poultry and compost heaps were systematically recorded in a 100 m buffer surrounding the kitchen gardens. Fresh burrow entries and fresh earth tumuli in kitchen gardens, respectively considered as an indicator for the presence of *Microtus* sp. and *Arvicola* sp. (Giraudoux et al., 1995; Quéré et al., 2000), were recorded at each sampling session. All of these variables were identified as ‘Food resources variables’. In addition, the presence/absence of vole habitats (i.e. pasture, meadow, forest edges and arable land) was considered in the 100 m buffer surrounding the kitchen garden, as well as the presence/absence of dairy farms that could attract foxes (Lucherini et al., 1995). These two variables were categorized as ‘Habitat variables’.

#### 2.3.3 Landscape composition surrounding the village and village clustering

Villages surrounded by dense vegetation and therefore offering safe resting locations are the most frequented by red foxes (Janko et al., 2012). This was considered as a potential driver of their faecal deposits in kitchen gardens. Landscape composition was described as a percentage of the type of cover found in the study areas, i.e. forest, arable land, hedges, grassland or built up. This percentage was estimated in the 5-km buffer surrounding the sampled village, using data from BD TOPO database (IGN, France) under QGIS version 1.8.0 (Quantum, 2013).

To reduce the number of variables for analyses, a hierarchical cluster was constructed, following a Principal Components Analysis (PCA), to group villages based on their land cover characteristics (see Lê et al., 2008 for the procedure). The PCA of the land cover variables was performed using the FactoMineR package (Husson et al., 2017). Covariates that explained the variability of village land cover were identified by estimating their contribution to the main reduced dimensions of the analysis. The most homogeneous land cover groups were determined by Kuiper's test (“v.test”, Kuiper, 1960) able to assess the contribution of each variable for each cluster by estimating the difference between the mean per cluster and the overall mean for each variable. The groups from this hierarchical clustering were used in further analyses through the ‘Village clustering variable’.

### 2.4 Factor analysis of faecal deposit

At least one carnivore scat was collected in 77% of the 192 sampled kitchen gardens. Based on qPCR analysis of the 1016 faeces collected, 58.7% of the copro-samples were from cats, 31.3% were from foxes and 9.8% were from dogs. Cat faeces, fox faeces and dog faeces were respectively found in 67%, 45% and 22% of the sampled kitchen gardens. More precisely, cat faeces were found in 52.5% of the 40 enclosed kitchen gardens, whereas only 17.5% included fox and 20% contained dog faeces. Cat faeces, fox faeces and dog faeces were found in 72%, 46% and 20% of the 92 partially open kitchen gardens and in 70%, 63% and 28% of the 40 open ones.

Because of the relatively small number of dog faecal samples, the following analysis was only conducted on deposits from cats and foxes. Furthermore, because the mean density of faeces was 11.85 times lower in the seven large cultivated areas devoted to market gardening than in the 185 kitchen gardens devoted to household consumption (0.002 faeces/m^2^ versus 0.023 faeces/m^2^ respectively), only the data from the 185 privately owned kitchen gardens were used for further analyses.

#### 2.4.1 Model fitting and evaluation

The effect of kitchen garden accessibility (‘Fence variable’ with three modalities: ‘Open’, ‘Enclosed’ and ‘Partially open’), food resource availability (including ‘Poultry’, ‘Compost’, ‘Fruit trees’, ‘Microtus’ and ‘Arvicola’ variables) and habitat (including ‘Pasture’, ‘Meadow’, ‘Arable land’ and ‘Farm’ variables) on the number of fox faeces (‘Fox faeces’ variable) and cat faeces (‘Cat faeces’ variable) per kitchen garden and per session was modelled.

A Generalized Linear Mixed effects Model (GLMM) was fitted using the glmmADMB function of the package “glmmadmb” under R statistical software (Skaug et al., 2013, R Core Team, 2017). ‘Fox faeces’ and ‘Cat faeces’ variables were found to be over-dispersed and were therefore modelled as negative binomial distributed dependent variables. The log-transformed value of the size of kitchen gardens (‘Size’ variable) was incorporated as an offset variable in the models since it differs between sampling units. Because ‘Fox faeces’, ‘Cat faeces’, ‘Arvicola’ and ‘Microtus’ variables were measured repeatedly in time, a random effect on the kitchen gardens was added into the model and took into account the within-garden correlation among observations. The effects of the year (‘Year’ variable) and months (‘Month’ variable) of the sampling were added as fixed effects. The village where the kitchen garden is located (‘Village’ variable) was also incorporated as a random effect since the number of villages was too large (N = 38) to incorporate as a covariate. Regarding the small number of replicates per group for random factors, only a random intercept, and not a random slope term, was added to the model.

The effects of all covariates in reducing the null model deviance was tested using the Likelihood Ratio test and model AIC comparison (ΔAIC; Zuur et al., 2009). The explanatory weight gained by adding a random intercept term for the in-kitchen garden and for the in-village variability was then assessed with the same metrics, keeping only the significant random effect term for further analysis. Model fit was evaluated by estimating the over-dispersion parameter. Finally, in order to investigate the presence of spatial autocorrelation in model residuals that could not have been explained by the model, the Moran index between kitchen gardens up to a distance of 5 km was computed; 5 km corresponding to the 95% quantile of the distances between kitchen gardens within the same villages. The null hypothesis (i.e. ‘no spatial correlation’) was tested using a normal approximation with the ‘correlog’ function of the ‘pgirmess’ package.

#### 2.4.2 Variable effects

The importance of each covariate was evaluated by estimating the loss of explained deviance induced by dropping each covariate from the models: the difference in AIC and a Likelihood Ratio test between the models “including” versus “excluding” each covariate were computed. The relative odds ratio (estimated as the exponential regression coefficients) and their 97.5% confidence interval were provided for each explanatory variable. For each covariate, one level was considered as the reference (model intercept) toward which the expected faeces count per unit area was compared. This reference level was set as ‘absence’ for the binary variables, and as ‘the one with the lowest observed faeces density’ for the categorical variables with >2 levels. March was set as a reference level for the ‘Month’ variable, Village cluster 3 fulfilled this function for the ‘Village cluster’ variable and 2016 took on the ‘Year’ variable, even if its effect could not be interpreted regarding the lower sampling effort for this year (a unique session and in Moselle only).

#### 2.4.3 Collinearity between covariates

Collinearity (i.e. high correlation) between covariates was documented using the Cramer's V statistic which provides an estimation of the strength of the associations. Moderate to strong associations (Cramer's V > 0.3) were observed between ‘Year’ and ‘Month’ (Cramer's V = 0.57), ‘Microtus’ and ‘Arvicola’ (Cramer's V = 0.44), ‘Village cluster’ and ‘Pastures’ (Cramer's V = 0.36). After model fitting, we checked if their effect changes when the influential covariates have been removed individually from the model.

## 3 Results

### 3.1 Village clustering

The first two dimensions of the Principal Component Analysis explained 61% of the variability between villages regarding the land-cover classes and were thus used for the clustering. The first dimension (x-axis) allows discriminating villages regarding the cover percentage of forests (x-axis contribution = 38%), arable lands (x-axis contribution = 26%) and buildings (x-axis contribution = 21%). The second dimension (y-axis) discriminated villages regarding mainly the cover percentage of grassland (x-axis contribution = 60%). The optimal level of discrimination allows classifying the villages in four clusters: Cluster 1 groups villages surrounded by forests and grassland (respectively v.test = 3.93 and v.test = 3.53; p < 0.001); Cluster 2 groups villages mainly surrounded by grassland (v.test = 4.67; p < 0.001); Cluster 3 groups villages mainly covered by buildings (v.test = 5.29; p < 0.001); Cluster 4 groups villages mainly surrounded by arable lands (v.test = 3.21; p < 0.001).

### 3.2 Drivers of fox faeces deposits

The covariates improved model predictions over the null model (Likelihood ratio test: deviance = 132; p-value < 2.2e−16; ΔAIC = 90). While adding a random effect on the kitchen garden improved model fit (Likelihood ratio test: deviance = 54; p-value ≤ 2.2e−13; ΔAIC = 52), an additional random effect on the Village did not add any further explanation of the data variability (Likelihood ratio test: deviance = 0.5; p-value = 0.476; ΔAIC = −1.5). Also, only the within kitchen garden variability was further considered as a random effect in the model. No over-dispersion was found in the residuals of the resulting full model (ratio = 0.7; Chi^2^ = 1032; p = 1). The model residuals were not found to be spatially autocorrelated given that none of the distance classes showed a significant Moran index (Appendix A).

**Fig. 2:**
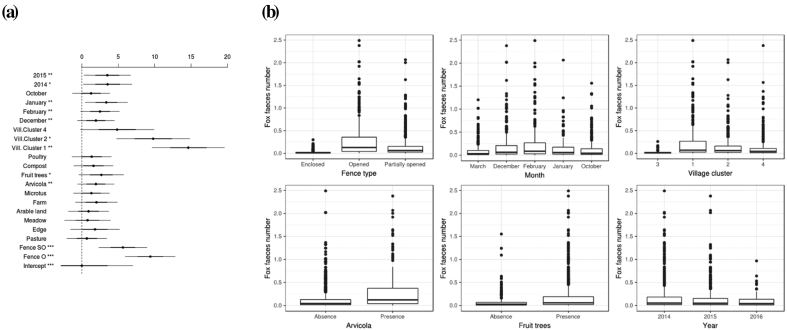
Outputs from the fox faeces deposit model. (a) Variable effects (odds ratio and their 97.5% confidence intervals) for the full model. The more fare away a variable is from the intercept, the highest is its effect on faeces deposit. (b). Expected number of fox faeces per kitchen garden predicted by the parsimonious model according to the different levels of each influential variables.

**Fig. 3:**
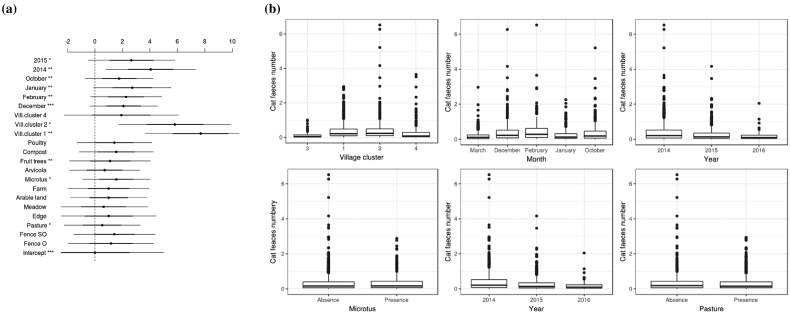
Outputs from the cat faeces deposit model. (a) Variable effects (odds ratio and their 97.5% confidence intervals) for the full model. The more fare away a variable is from the intercept, the highest is its effect on faeces deposit. (b). Expected number of cat faeces per kitchen garden predicted by the parsimonious model according to the different levels of each influential variables.

From the backward variable selection, ‘Fence’ ‘Month’, ‘Village cluster’, ‘Arvicola’, ‘Fruit trees’ and “Year”, in order of importance, were selected as explanatory variables of fox faeces deposits (Table 1). The expected number of fox faeces found per kitchen garden and by session differed according to the ‘Fence’ variable (Fig. 2): compared to enclosed kitchen gardens, they were was nine times larger in open kitchen gardens (Odds ratio OR) = 9.4 (CI = 3.27–27.98) and about five times larger in partially open kitchen gardens (OR = 5.64; CI = 2.10–15.12). Also, the number of fox faeces was the largest in January (Fig. 2), increasing by a factor of 3.4 (CI = 1.61–7.05) in comparison to March. The expected number of fox faeces was larger in the kitchen gardens located in the Village cluster 1 (Fig. 2), i.e. in villages mainly surrounded by forests and grassland (OR = 14.62; CI = 2.46–87.01), and in Village cluster 2, i.e. in village surrounded by grassland (OR = 9.8; CI = 1.61–59.64) in comparison to those mainly covered by buildings (Village cluster 3). The number of fox faeces per kitchen garden did not differ between Village cluster 4 (surrounded by arable land) and other village clusters (Fig. 2). The number of fox faeces increased by a factor of 1.93 (CI = 1.24–3.02) with the presence of *Arvicola* sp. and by a factor of 2.68 (CI = 1.15–6.22) when fruit trees are present in the immediate vicinity of the kitchen garden (Fig. 2). The number of fox faecal deposits were also three times greater than in 2015 (OR = 3.50; CI = 1.40–8.79) and in 2014 (OR = 3.55; CI = 1.31–9.64) than in 2016 (Fig. 3). Finally, the presence of a farm in the vicinity of the kitchen garden doubled the chances of finding fox faeces (OR = 2; CI = 0.98–4.10).

**Table 1:**
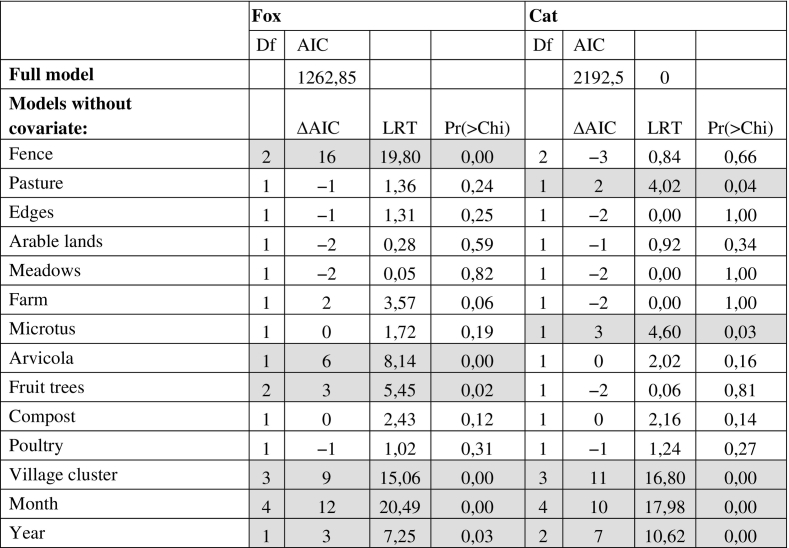
Variables backward selection for the fox and cat faeces deposit models. The degrees of freedom (Df), the difference in AIC between the full model and the models excluding each covariate (ΔAIC), the Likelihood ratio test statistic (LRT, Chi-squared test) and its *p*-value (Pr > Chi) are provided for the models that excludes each covariate one-by-one.

When influential covariates have been removed one by one, ‘Microtus’ had an effect when ‘Arvicola’ was removed (ΔAIC = 5; LRT = 7.332; Pr(>Chi) = 0.007; OR = 1.7[1.16–2.49]).

### 3.3 Drivers of cat faeces deposits

Similar to the model for fox faecal deposits, the covariates improved the model predictions for cat faecal deposit over the null model (Likelihood ratio test: deviance = 92.8; p-value = 5.28e−11; ΔAIC = 51). Adding a random effect on the kitchen garden improved model fit (Likelihood ratio test: deviance = 132; p-value < 2.2e−16; ΔAIC = 130) while an additional random effect on the Village did not add any further explanation to the kitchen garden random effect (Likelihood ratio test: deviance = 0; p-value = 1; ΔAIC = −2). Consequently, only the within kitchen garden variability was further considered as a random effect. No over-dispersion in residuals of the full model was found (ratio = 0.65; Chi^2^ = 1456; p = 1). Also, the model residuals were not found to be spatially autocorrelated (Appendix A).

From the backward variable selection, “Village cluster”, “Month”, “Year”, “Microtus” and “Pasture” were selected as explanatory variables of cat faecal deposits (Table 1). The effect of the “Village cluster” was the most important (Fig. 3): as it was the case for the fox, cat faeceal density was the largest in the villages surrounded by forests and grassland (Village cluster 1) and then in villages surrounded by grassland (Village cluster 2), increasing by a factor of 7.7 (CI = 1.94–30.62) and 5.8 (CI = 1.43–23.61) in those clusters respectively, in comparison to the Village cluster 3 (surrounded by buildings). The number of cat faeces per kitchen garden did not differ in Village cluster 4 (surrounded by arable land) nor from other village clusters (Fig. 3). The expected cat faecal density per kitchen garden was at a maximum in January, increasing by a factor of 2.72 (CI = 1.37–5.35) in comparison with March. It was also higher in February (OR = 2.25; CI = 1.35–3.76) and December (OR = 2.08; CI = 1.37–3.16) than in March (Fig. 3). Cat faeceal deposits differed between years: it was four times larger in 2014 (OR = 4.06; CI = 1.55–10.64) and 3 times larger in 2015 (OR = 2.66; CI = 1.08–6.51), than in 2016 (Fig. 3). It was also positively correlated with ‘Microtus’, increasing by a factor of 1.56 [1.04–2.34] when the species was present (Fig. 3). Finally, cat faecal deposits were slightly fewer in kitchen garden surrounded by pastures than in other kitchen gardens (OR = 0.52; CI = 0.28–0.99).

When influential covariates were individually removed one from the model, the effect of ‘Pasture’ turned out to be not significant when ‘Microtus’ (LRT = 3.30; Pr(>Chi) = 0.069) and ‘Village cluster’ (LRT = 0.76; Pr(>Chi) = 0.383) variables were removed.

## 4 Discussion

By analysing the faecal deposit drivers in kitchen gardens, this study identifies the factors that contribute to the in-field contamination of fruit and vegetables with canid and felid foodborne parasites in a particular location. It provides information on guiding species-specific preventive measures that considers carnivores in the context of a foodborne zoonoses contamination risk.

At least one carnivore scat was found in 77% of the 192 kitchen gardens prospected, confirming Poulle et al. (2017) and Bastien et al. (2018) findings that numerous kitchen gardens located in north-eastern France are a likely spot for carnivore faecal deposits and, consequently, at-risk of foodborne parasite transmission from contaminated soils or fruit and vegetables to humans. Dog faeces accounted for <10% of those collected and were found in only 22% of the kitchen gardens prospected, probably because there were no stray dogs and few pet dogs in the study area. Despite this relatively low abundance, the deposits of dog faeces in kitchen gardens should be taken into account since dog faeces are known to potentially spread *E. multilocularis* in the human environment (Vaniscotte et al., 2011; Umhang et al., 2014), but also *Echinococcus granulosus* sensu lato (Acosta-Jamett et al., 2010; Van Kesteren et al., 2013; Mastin et al., 2011) and *Toxocara canis* (Blaszkowska et al., 2011; Dubná et al., 2007; Rubel and Wisnivesky, 2005). Dog faeces are also identified as potential source of *E. coli* and *Salmonella* contamination of lettuce (Jay-Russell et al., 2014).

The spring thaw and the beginning of vegetable plot preparation, increases the rate of faecal decomposition and may explain why a lower number of cat and fox faeces were found during the March sessions. Furthermore, because of the typical cat behaviour of burying their faeces, this detection rate was probably lower than those of canids during any session. However, despite these potential underestimations, cat faeces represented more than half of the 1016 collected faeces, and at least one cat scat was found in almost 2/3 of the 192 prospected kitchen gardens. The presence of cat faeces was positively correlated with the presence of *Microtus* sp. that is one of the main cat preys in the study area (Forin-Wiart, 2014). Additionally, the high density of the rural cat population, the cat's attraction toward loose soil suitable to cover faeces, their low fear of humans and their ability to pass through or to climb over fences, may explain the high occurrence and large distribution of cat faeces in kitchen gardens. The consumption of unwashed, raw fruit and vegetables has been identified as a transmission pathway for human toxoplasmosis (Liu et al., 2009; Alvarado-Esquivel et al., 2011). Because a single cat in first infection can excrete in their faeces millions of *T. gondii* oocysts (Dubey, 2004) that in turn can survive at least 2 years in low temperatures and very humid conditions (Lélu et al., 2012), the deposit of kitten faeces (mainly responsible for environmental contamination) in kitchen gardens may be a concern regarding human infection by this parasite.

The main results of this study concern fox faeces, representing >30% of the 1016 collected faeces and found in 53% of the 152 open or partially open kitchen gardens. The abundance of fox faeces in kitchen gardens located in high AE endemic areas is concerning with regard to *E. multilocularis* transmission to humans. Likewise, to a lesser extent, for *T. canis* transmission as this parasite is common in the European fox populations (Cerbo et al., 2008; Criado-Fornelio et al., 2000; Stuart et al., 2013). *Toxocara* sp. DNA was detected in 12% of the 70 fox faeces collected during autumn-winter 2012 and 2013 in 94 kitchen gardens of the study area (Poulle et al., 2017).

This study demonstrates that a fence or wall of at least 1.2 m in height is associated with considerably reduction fox faecal deposits in kitchen gardens. It also provides evidence that the landscape composition surrounding the village is a driver of fox faecal deposits; there are fewer deposits on urbanised and cultivated land than on land dominated by forests and grassland. The proximity of a farm, where barns can be used as resting and breeding sites by foxes (Sidorovich, 2013), and the presence of fruit trees were also found to be a predisposing factor for fox faecal deposits in kitchen gardens. Therefore, fencing should be strongly encouraged for kitchen gardens located in AE endemic areas, especially those surrounded by forest and grassland and/or near fruit trees or a farm. In addition, fencing should be regularly inspected to repair any gaps and the gate should be kept shut at all times to deter occasional canid intrusion. If fencing the whole garden is not feasible, the gardener should be encouraged to delineate and fence off the area devoted to the most at-risk crops, meaning leafy greens, edible herbs or strawberries that grow close to the ground and are often eaten raw. The cultivation of at-risk home-grown vegetables in upright window boxes or in a greenhouse with a 1 m high barrier to restrict access may also be encouraged.

Preventing foodborne parasite contamination of large fields used as market gardens is obviously more challenging. In this study, the density of fox, cat and dog faecal appeared lower in these locations than in the smaller kitchen gardens but it was not null. In the study area, all fresh produce grown outside cannot be considered as “free from foodborne parasites”. Consequently, technical and financial assistance/support for suitable fencing should be provided by public authorities to market gardeners in order to reduce the likelihood of parasitic contamination of their produce, especially when fields are located in AE endemic area. The 0.8% *T. gondii* contamination of ready-to-eat salads grown in Southern Italy (Caradonna et al., 2017) suggests that the problem of carnivore foodborne parasite contamination of fresh produce grown outside is not limited to these areas.

Rodent trapping in kitchen gardens and crop growing should also be encouraged to reduce fox and cat deposits as this study shows, the presence of *Arvicola* sp. and *Microtus* sp. are drivers for fox and cat faecal deposits. The presence of *Microtus* sp. was also identified as a driver for fox faecal deposits even if our analysis showed its effect is partially hidden by those of *Arvicola* sp. Targeted mitigation strategies specifically addressing produce safety and the risks of parasitic contamination with carnivore faeces should not only include advice on fencing and rodent trapping but also highlight the risk to human health. Proactive information focusing on a target group, as recommended by Hegglin et al. (2008) should avoid known hazardous behaviour such as allowing carnivores free access to kitchen gardens, using the enclosed kitchen gardens as a dog pen or using cat litter as manure. Here, the target group would be growers and owners of kitchen gardens.

## Figures and Tables

**Figure:**